# Factors associated with pain and functional impairment five years after total knee arthroplasty: a prospective observational study

**DOI:** 10.1186/s12891-023-07125-y

**Published:** 2024-01-02

**Authors:** Unni Olsen, Vibeke Bull Sellevold, Caryl L. Gay, Arild Aamodt, Anners Lerdal, Milada Hagen, Alfhild Dihle, Maren Falch Lindberg

**Affiliations:** 1 Department of Public Health Science, Institute of Health and Society, Faculty of Medicine, University of Oslo, Oslo, Norway; 2 Department of Orthopaedic Surgery, Lovisenberg Diaconal Hospital, PB 4970 Nydalen, Oslo, 0440 Norway; 3 Lovisenberg Diaconal University College, Oslo, Norway; 4 Faculty of Health Sciences, Department of Nursing and Health Promotion, Oslo Metropolitan University, Oslo, Norway; 5 Department of Family Health Care Nursing, University of California, San Francisco, USA; 6 Research Department, Lovisenberg Diaconal Hospital, Oslo, Norway; 7 Department of Interdisciplinary Health Sciences, Institute of Health and Society, Faculty of Medicine, University of Oslo, Oslo, Norway

**Keywords:** Knee arthroplasty, Pain, Chronic pain, Function, Osteoarthritis, Prognosis

## Abstract

**Background:**

Few studies have evaluated the associations between preoperative factors and pain and physical function outcomes after total knee arthroplasty (TKA) from a mid-term perspective. Identification of such factors is important for optimizing outcomes following surgery. Thus, we examined the associations between selected preoperative factors and moderate to severe pain and pain-related functional impairment as measured using the Brief Pain Inventory (BPI), five years after TKA in patients with knee osteoarthritis.

**Methods:**

In this prospective observational study, all patients scheduled for primary unilateral TKA for osteoarthritis were consecutively recruited. Preoperative factors identified from previous meta-analyses were included to assess their associations with pain severity and pain-related functional impairment five years after TKA. Pain severity was the primary outcome, while pain-related functional impairment was the secondary outcome. The BPI was used to evaluate outcomes five years post-TKA. Statistically significant factors from univariate regressions were entered into a multiple logistic regression model to identify those with the strongest associations with pain severity or pain-related functional impairment five years after TKA.

**Results:**

A total of 136 patients were included, with a mean age of 67.7 years (SD 9.2) and a majority being female (68%). More severe preoperative pain (OR = 1.34, 95% CI [1.03 to 1.74]), more painful sites (OR = 1.28., 95% CI [1.01 to 1.63]), and more severe anxiety symptoms (OR = 1.14., 95% CI [1.01 to 1.28]) were associated with *increased* likelihood of moderate to severe pain five years after TKA surgery, while more severe osteoarthritis (OR = 0.13, 95% CI [0.03 to 0.61]) was associated with *reduced* likelihood of moderate to severe pain five years after TKA. More severe anxiety symptoms (OR = 1.25, 95% CI [1.08 to 1.46]) were also associated with *increased* likelihood of moderate to severe pain-related functional impairment five years after surgery, while male sex (OR = 0.23, 95% CI [0.05 to 0.98]) was associated with *reduced* likelihood of pain-related functional impairment five years after surgery.

**Conclusion:**

The identified preoperative factors should be included in larger prognostic studies evaluating the associations between preoperative factors and mid-term pain severity and physical function outcomes after TKA surgery.

**Supplementary Information:**

The online version contains supplementary material available at 10.1186/s12891-023-07125-y.

## Background

Total knee arthroplasty (TKA) is a widely accepted and cost-effective surgical procedure intervention for end-stage knee osteoarthritis [1, 2]. However, it has been estimated that one in five patients experience persistent knee pain and limited functional improvement following TKA [3, 4]. Most of the improvement in pain and physical function levels occurs and plateaus during the first year after TKA, followed by smaller gains or even worsening in pain and physical function levels thereafter [3, 5–10]. Ongoing pain and impaired physical function not only necessitate the consideration of revision surgery but also impose significant burdens on affected individuals [11, 12] and substantial demands on healthcare [11–13]. By better understanding the factors associated with these poor outcomes, we aim to enhance postoperative care and optimize long-term outcomes for patients undergoing TKA.

Prior meta-analyses of studies reporting outcomes from one to five years after TKA, have identified preoperative pain catastrophizing, mental health, pain, number of painful sites, and severity of osteoarthritis as being associated with persistent pain [14–17], while preoperative physical function, mental health, body mass index (BMI), and severity of osteoarthritis are associated with persistent impairment in physical function [14, 16, 18]. However, few studies have evaluated factors associated with the mid-term outcomes (five years) after TKA and their results have been conflicting [3, 10, 19–21]. Thus, uncertainty remains regarding the factors correlated with pain and physical function beyond one year after TKA.

To address this knowledge gap, we selected preoperative factors with the strongest evidence of association with pain and physical function in prior meta-analyses of one to five years outcomes [14–18] and evaluated the strength of their associations with pain and pain-related functional impairment five years after TKA. We hypothesized that preoperative pain, number of painful sites, anxiety, and severity of osteoarthritis are associated with moderate to severe pain five years after TKA. Additionally, we hypothesized that preoperative pain-related functional impairment, BMI, anxiety, and severity of osteoarthritis are associated with moderate to severe pain-related functional impairment five years after TKA.

## Methods

### Study design

This study is a five-year follow-up study stemming from a longitudinal prospective study on pain, functioning and quality of life completed in 2014 [6]. Methodological details are described in a prior report from the same research group [6]. Reporting of the current analysis is in accordance with the **“**STrengthening the Reporting of OBservational studies in Epidemiology” (STROBE) initiative and checklist [22], as detailed in Additional information. The study was approved by the Regional Medical Research Ethics Committee of Health South-East of Norway (no. 2011/1755).

### Study sample and procedures

Inclusion and baseline data collection took place between October 2012 to August 2013. Data collection for the five-year follow-up was performed from October 2017 until December 2018. Patients were consecutively recruited and included in the original study if they were scheduled for primary unilateral TKA for osteoarthritis at Lovisenberg Diaconal Hospital in Oslo, Norway, were 18 years or older, were able to read, write and understand Norwegian. Patients were excluded if they underwent an unicompartmental knee arthroplasty or a revision surgery or had a diagnosis of dementia. Patients completed a baseline questionnaire prior to surgery that included sociodemographic characteristics and preoperative symptoms and clinical factors. Data on BMI, comorbidities, American Society of Anaesthesiologists’ physical status classification (ASA) [23], medication and osteoarthritis severity were obtained from medical records. Anaesthesiologists performed the ASA assessments prior to surgery.

The surgeries were performed by a team of ten to fifteen surgeons. The same posterior cruciate-retaining fixed modular-bearing implant (The Profix Total Knee System, Smith and Nephew, Memphis, USA) was used in all surgeries, and patients were treated according to a standardized protocol with regard to anaesthesia, surgical procedures, pain management, postoperative mobilisation and physical therapy, as previously described in detail [6, 24].

For the current five-year follow-up study, all participants from the original study were invited to participate. Those who agreed to participate signed a new consent form. Most patients were scheduled for a five-year appointment at the hospital and were given the option to complete the questionnaires on iPads on-site or on paper at home. Those who completed paper questionnaires returned them by mail in pre-paid sealed envelopes. Patients who did not complete the questionnaire received one reminder either by telephone or mail.

### Measures

The Brief Pain Inventory (BPI) was used to measure pain severity (primary outcome) and pain-related functional impairment (secondary outcome). The BPI consists of four items to measure pain severity (pain right now, as well as average, worst, and least pain in the past 24 h). Additionally, the BPI includes seven items to rate pain interference with function (general activity, walking, work, mood, enjoyment of life, relations with others, and sleep). Furthermore, the BPI incorporates a body map to determine the number and location of painful sites. The BPI is scored on a 0–10 numerical rating scale (NRS), from no pain or no interference with function to pain as bad as you can imagine or total interference with function [25]. We used the average pain item from the BPI to measure pain five years after TKA, and as recommended by IMMPACT panel [26], we used the BPI pain interference with function index to measure pain-related functional impairment. We followed the recommendations in the BPI user guide and calculated the mean of the seven interference items, as long as at least four of the seven items were answered [25]. The Norwegian version of the BPI has shown acceptable consistency, reliability, construct validity and responsiveness in the assessment of pain in a sample of patients with osteoarthritis waiting for total hip arthroplasty [27]. Optimal cut-points for average pain ratings in TKA patients are none/mild (0–3), moderate (4–6) and severe (7–10) [28, 29]. Cut-off values points for the BPI pain interference index are not established for patients undergoing TKA. We therefore used the cut-points identified in a study of patients with low back pain: none/mild (0–3), moderate (4–5) and severe (6–10) [30]. BPI scores for pain and pain interference with function index five years after surgery were dichotomized into none to mild (0–3) and moderate to severe (4–10) for analysis.

### Measurement of selected preoperative variables

The selected preoperative variables hypothesized to be associated with pain and function at five-year follow-up are shown in Table 1. The factors were selected based on evidence from prior meta-analyses [14–18]. Factors in the meta-analyses that had weak evidence of association with pain and function were not selected and are listed in the footnote of Table 1.

**Table 1:** Selected preoperative factors identified in prior meta-analyses

	Pain	Physical function
Possible associated factor	Pain [15, 17]	Functional impairment [18]
Possible associated factor	Osteoarthritis severity [16, 17]	Osteoarthritis severity [16, 18]
Possible associated factor	Anxiety [14, 15]	Anxiety [14]
Possible associated factor	Number painful sites [15, 17]	Body Mass Index (kg/m^2^) [18]
Control factor	Age	Age
Control factor	Sex	Sex
Control factor	Comorbidity	Comorbidity

Note: The following factors were included in prior meta-analyses [14–18] but were excluded from this study due to weak evidence of their association with pain and function outcomes: age, comorbidities, depression, education, function, gender, patella resurfacing, social support, weight [15]

The BPI was used to measure patients’ preoperative average pain severity, and their number of pain locations was measured using the BPI body diagram. Osteoarthritis severity was determined by classifying the patients’ preoperative radiographs using the Kellgren-Lawrence (K-L) classification system [31]. An experienced musculoskeletal radiologist and an orthopedic surgeon who were blinded to the clinical data evaluated the radiographs. The K-L grades range from 0 to 4, with higher grades indicating more severe osteoarthritis. In our analysis, we dichotomized the K-L grade used in the logistic regression model into mild to moderate (K-L grades 2 or 3) or severe osteoarthritis (K-L grade 4) using the same cut-off as a previous study [32]. Symptoms of anxiety and depression were measured with the Hospital Anxiety and Depression Scale (HADS), which consists of seven items for measuring anxiety and seven items for depression [33]. Scores for each subscale range from 0 to 21, with higher scores indicating more symptoms of depression or anxiety. A dichotomized HADS score was used in the analysis, HADS score < 8 indicating less symptoms, and a score ≥ 8 indicating more symptoms [34]. The tool has been evaluated in a large Norwegian population study and was found to have excellent psychometric properties [35]. BMI was calculated as kg/m^2^. Comorbidities were counted and divided into four categories (0, 1, 2, ≥ 3).

### Statistical analyses

Our study used a longitudinal design and aimed to identify possible prognostic factors, thus our sample size consideration was only related to precision of our estimates. To ensure sufficient statistical power and reliability of the findings, we aimed to include at least 100 individuals in our study. Further, as there was only a limited number of patients in the no/mild pain or interference group we aimed to include at most four covariates in our multivariable model to avoid overfitting.

Summary statistics for the sample characteristics were calculated and presented as means with standard deviations (SD) for continuous variables and frequencies and proportions for categorical variables. Data were checked for missing values, and baseline characteristics for those who were lost to follow-up were compared to those who remained in the study at five years. No imputation of data was performed, as we did not have sufficient statistical power to perform any model-based imputation.

For analyses of associations, we dichotomized the pain and pain interference with function outcomes (dependent variables) as < 4 (none or mild, coded 0) or ≥ 4 (moderate to severe, coded 1). For the preoperative factors (independent variables), sex (male or female), and K-L grade (2–3 or 4) were dichotomous, while all others were treated as continuous. We first examined univariate associations using a logistic regression model between each of the selected preoperative factors identified in prior meta-analyses and the pain and pain interference with function outcomes. In addition, we evaluated age, comorbidity and sex in univariate models. Variables that were statistically significant in univariate analyses (*p* < 0.05) were entered into a multivariable logistic regression model using a conditional backward selection method, as recommended for evaluating prognostic factors [36]. The factor with the least significant *p*-value (≥ 0.10) was removed from the model, followed by a refit with the remaining factors in the next step. This process was repeated until all the included variables were statistically significant (*p* ≤ 0.05).

We investigated the effect of extreme observations (outliers), removed these, and performed sensitivity analyses. To evaluate the logistic regression model assumptions, we assessed linearity between independent variables and the log odds of the dependent variable. Multicollinearity was examined by analysing correlation coefficients between independent variables, and if two variables had a correlation of r ≥ 0.7, one of them was excluded [37]. All analyses were considered exploratory and no correction for multiple testing was done. *P*-values < 0.05 were considered statistically significant and all tests were two-sided.

Data analyses were performed using SPSS for Windows, version 28 (IBM Corp, Armonk, NY).

## Results

### Demographic and clinical characteristics

**Fig. 1:**
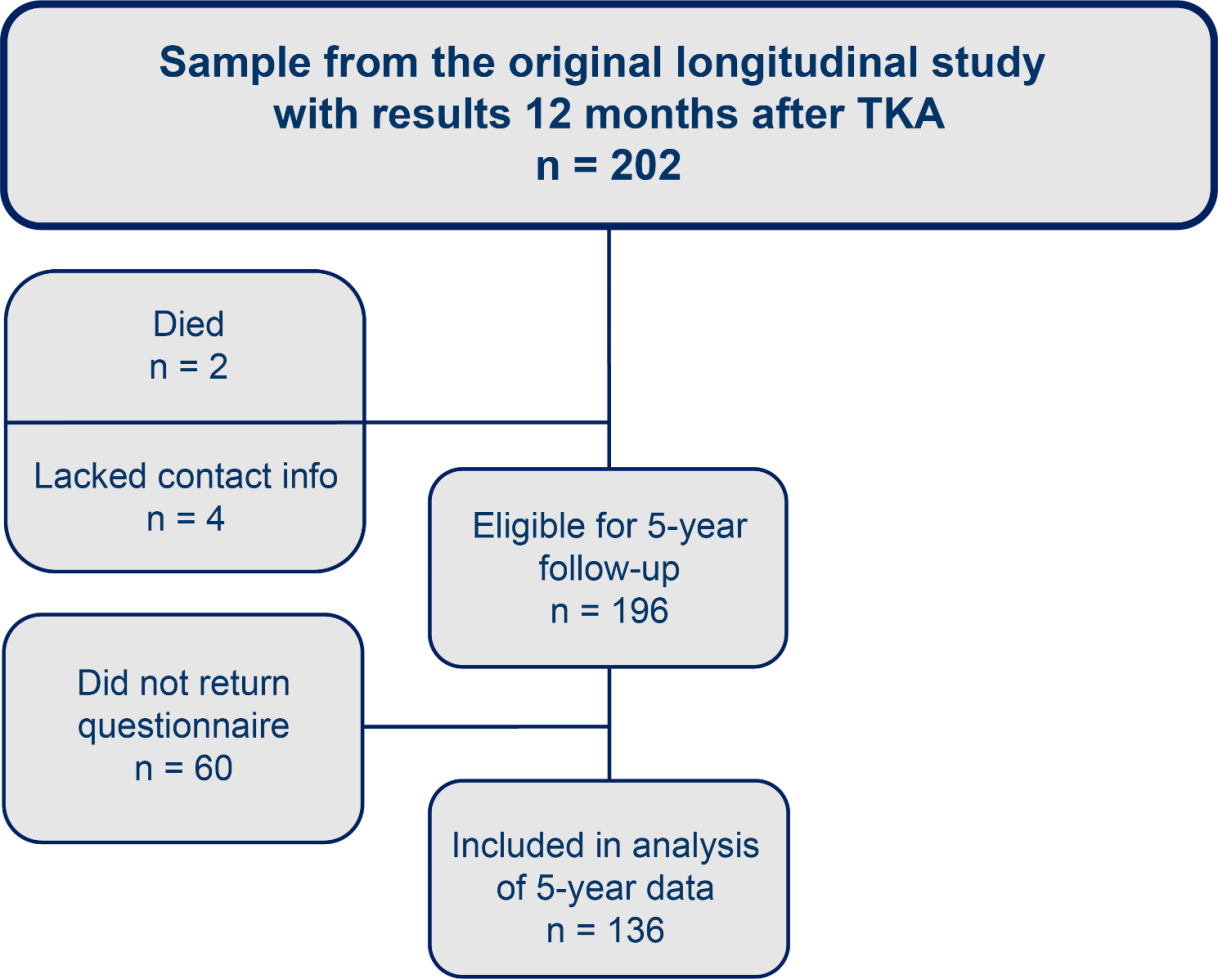
Flowchart of study participants

In the original longitudinal study, and as shown in Fig. 1, 245 patients were invited to participate, of which 202 were included. Details regarding the enrolment process for the original study have been previously described [6]. For the current study, we considered all 202 patients from the original study eligible for inclusion, but as two patients had died, and four had no available contact information, we invited 196 patients to participate in this follow-up study. Of these, 60 did not return the questionnaire, leaving 136 (67%) consenting patients for inclusion in the final analysis. Baseline characteristics of the cohort are presented in Table 2.

**Table 2:** Preoperative demographic and clinical characteristics (N = 136)

Characteristics	n	Statistics	
**Demographic characteristics**		**Mean**	***SD***
Age in years	136	67.7	9.1
		**n**	**%**
Sex	136		
Male		44	32
Female		92	68
Cohabitation status	136		
Lives alone		53	39
Married/partnered		83	61
Education	135		
High school or lower		64	47
College/university		72	53
**Clinical characteristics**		**Mean**	***SD***
BMI	136	28.6	4.2
Number of comorbidities	136	1.2	1.0
ASA score (1–3)	136	2.0	0.5
Osteoarthritis severity	135		
Mild to moderate (K-L grade ≤ 3)		107	79
Severe (K-L grade = 4)		28	21
Number of painful sites	136	2.1	1.8
BPI pain ratings on 0–10 scale			
Worst pain	136	5.3	2.1
Average pain	135	5.2	1.8
Pain interference with function index	136	4.4	2.0
BPI pain categories (dichotomized)		**n**	**%**
Average pain	135		
None/mild (< 4)		28	21
Moderate/severe (≥ 4)		107	79
Pain interference with function index	136		
None/mild (< 4)		58	43
Moderate/severe (≥ 4)		78	57
HADS anxiety score	129		
Low (< 8)		99	77
High (≥ 8)		30	23
HADS depression score	130		
Low (< 8)		111	85
High (≥ 8)		19	15

Abbreviations: ASA, American Society of Anaesthesiologists’ physical status classification; BPI, Brief Pain Inventory; BMI, Body Mass Index; K-L, Kellgren Lawrence; HADS, Hospital Anxiety and Depression Scale

In short, the mean age of this sample was 67.7 (SD 9.1) years, and most participants were female (68%) and lived with a partner (61%). Comparing patients who were included in the five-year follow-up with those who were not, there were no statistically significant differences in age (*p* = 0.26), sex (*p* = 0.87), ASA classification (*p* = 0.36), number of comorbidities (*p* = 0.17), number of painful sites (*p* = 0.14), average pain (*p* = 0.11), pain-related functional impairment (*p* = 0.88) or symptoms of anxiety (*p* = 0.85) or depression (*p* = 0.25). Patients included in the five-year follow-up sample had statistically lower preoperative BMI (mean 28.6, SD 4.2) than those who were not included (mean 30.3, SD 5.6) (*p* = 0.03), but the effect size was small (Cohen’s d = 0.3).

### Pain severity

As shown in Tables 2 and 3, the average pain score declined from a mean value of 5.2 (SD 1.8) preoperatively to 2.7 (SD 2.3) five years after surgery (*p* = 0.17). The vast majority of patients (79%) reported moderate to severe pain (BPI ≥ 4) prior to surgery and this proportion decreased to about one third (32%) five years after surgery.

**Table 3:** Descriptions of pain and functional outcome variables at five-year follow-up

Outcome variables	n	Statistics	
		**Mean**	***SD***
BPI average pain rating (0–10 scale)	136	2.7	2.3
BPI pain interference with function index (0–10 scale)	135	1.9	2.1
		**n**	**%**
BPI average pain rating (dichotomized)	136		
None/mild (< 4)		92	68
Moderate/severe (≥ 4)		44	32
BPI pain interference with function index (dichotomized)	135		
None/mild (< 4)		111	82
Moderate/severe (≥ 4)		24	18

Abbreviation: BPI, Brief Pain Inventory

As shown in Table 4, univariate logistic regression analyses revealed significant associations between pain five years after TKA and the following preoperative factors: pain, anxiety symptoms, radiographic osteoarthritis, painful sites and sex. Male sex and less severe radiographic osteoarthritis were associated with less pain. In the final multivariable regression model (Table 4), higher preoperative pain was the strongest prognostic factor for moderate to severe postoperative pain five years after TKA. For each 1-point increase in preoperative average pain rating, the odds for moderate to severe pain at five years increased by 34%, controlling for all other variables. Each additional painful site patients reported preoperatively increased the odds of having moderate to severe pain by 28%. In addition, each 1-point increase in the patient’s HADS anxiety score was associated with 14% higher odds for reporting moderate to severe pain five years after surgery. Those with severe radiological osteoarthritis had 87% lower odds for experiencing moderate to severe pain at five years, compared to those with moderate osteoarthritis. Sex was no longer associated with moderate to severe pain in the multivariable analysis and was therefore not retained in the final model (step 2).

**Table 4:** Associations between preoperative factors and moderate to severe average pain at 5-year follow-up

Preoperative factors	Univariate logistic regression				Multivariable logistic regression			
	OR	95% CI		*p* value	OR	95% CI		*p* value
K-L grade 4 (vs. ≤ 3)	0.12	0.03	0.53	0.005	0.13	0.03	0.61	0.010
Anxiety symptoms	1.19	1.07	1.33	0.002	1.14	1.01	1.28	0.035
Average pain	1.36	1.09	1.69	0.005	1.34	1.03	1.74	0.028
Number of painful sites	1.26	1.03	1.54	0.024	1.28	1.01	1.63	0.045
Age	1.01	0.96	1.04	0.847				
Number of comorbidities	1.31	0.93	1.84	0.122				
Male sex (female reference)	0.42	0.18	0.97	0.043				

Abbreviations: K-L grade, Kellgren Lawrence grade

### Pain-related functional impairment

For the secondary outcome, pain-related functional impairment (Tables 2 and 3), the mean score improved from 4.4 (SD 2.0) before surgery to 1.9 (SD 2.1) five years after surgery (*p* = 0.57). Prior to surgery, 78 (57%) patients had moderate to severe pain-related functional impairment based on their pain interference with function index, and this proportion declined to 24 (18%) patients five years after surgery.

The univariate logistic regression analyses revealed significant associations between pain-related functional impairment five years after TKA and the following preoperative factors: sex, anxiety symptoms and preoperative pain interference with function (Table 5). Male sex was associated with less pain-related functional impairment. When all variables that were statistically significant in univariate analysis were entered into the multiple logistic regression model, male sex and anxiety remained independent prognostic factors for pain-related functional impairment. Each 1-point increase in the patient’s preoperative anxiety score was associated with 25% higher odds for having moderate to severe pain-related functional impairment five years after TKA. Males were 77% less likely than females to have moderate to severe pain-related functional impairment. The removal of outliers did not substantially alter the overall results of the study.

**Table 5:** Associations between preoperative factors and pain-related functional impairment at 5-year follow-up

Preoperative factors	Univariate logistic regression				Multivariable logistic regression			
	OR	95% CI		*p* value	OR	95% CI		*p* value
Age	0.97	0.93	1.02	0.298				
Number comorbidities	1.17	0.78	1.76	0.434				
BMI	0.98	0.88	1.09	0.704				
K-L grade 4 (vs. ≤ 3)	0.14	0.02	1.09	0.061				
Male sex (female reference)	0.24	0.07	0.87	0.029	0.23	0.05	0.98	0.047
Anxiety symptoms	1.30	1.13	1.50	< 0.001	1.25	1.08	1.46	0.003
Pain interference function	1.45	1.14	1.83	0.002	1.20	0.90	1.60	0.20

Abbreviations: K-L grade, Kellgren Lawrence grade; BMI, body mass index

## Discussion

To our knowledge, this is the first study to investigate evidence-based preoperative prognostic factors’ associations with moderate to severe pain and pain-related functional impairment five years after TKA. Our findings indicate that all the preoperative factors identified in previous meta-analyses for pain, such as preoperative pain, painful sites, anxiety symptoms, and osteoarthritis severity, were also significantly associated with mid-term pain outcomes five years following TKA [14–17]. However, the same was not true for physical function, as BMI, preoperative functional impairment, and severity of osteoarthritis, which were identified as preoperative factors in prior meta-analyses, were not significantly correlated with physical function five years after TKA [16, 18]. Notably, a considerable proportion of the patients reported moderate to severe pain (32%), and pain-related functional impairment (18%) five years after TKA. Our findings largely confirm and clarify that certain factors have enduring effects on pain and physical function outcomes. These findings also contribute to addressing the gap in knowledge on the recovery course five-years after TKA.

Interestingly, more anxiety symptoms were a prognostic factor for both moderate to severe pain and moderate to severe pain-related functional impairment. Consistent with our findings, results from a registry study (Mayo Clinic Total Joint Registry) indicated that more severe anxiety increased the odds for more severe pain, but also for impaired physical function years after TKA [20]. In another prospective study, no correlations were identified between preoperative anxiety symptoms and pain or physical function outcomes at five years [21]. These conflicting results may be due to the low follow-up rate of 29% at five years in the latter study. High preoperative pain levels in OA patients have been correlated with higher levels of anxiety [38]. If untreated, anxiety and pain catastrophizing may hamper surgical outcomes as problems with pain catastrophizing, avoidance and worrying may complicate the recovery process for these patients [14, 39–41]. While the presence of anxiety symptoms should not be used as a criterion for patient selection, identifying patients at high risk before surgery and developing effective targeted psychological interventions that facilitate recovery after TKA may be important as such approaches are still lacking [42, 43].

Among all factors, higher preoperative pain had the strongest association with moderate to severe pain five years after TKA surgery. Our results for the five-year outcome are supported by results from previous well-conducted systematic reviews and meta-analyses [15, 17, 44]. Furthermore, we found that more painful sites before surgery were associated with moderate to severe pain five years after surgery, which is in line with findings from other studies [15, 45, 46]. Fibromyalgia is a disorder characterized by multiple pain sites, and findings from Brummet et al. [46] indicate that patients with this condition have less improvement than patients with fewer pain sites.

We also found that more severe radiographic osteoarthritis was associated with less pain five years after surgery, which aligns with findings from recent systematic reviews and meta-analyses [16, 17]. Klasan et al. [47] identified a subgroup of patients that had a combination of high preoperative pain and less severe osteoarthritis and were more likely to have higher levels of pain one year after TKA. The authors suggested that the aetiology of pain in this subgroup is multifaceted and more independent of osteoarthritis-specific pathology, highlighting the complex pain mechanism after surgery. Patients with severe radiographic osteoarthritis might be more affected by disease symptoms than patients with less severe osteoarthritis, potentially explaining why they tend to benefit more from TKA surgery than patients with milder osteoarthritis [48]. It is important to note that patients with less severe osteoarthritis can still obtain significant benefits from TKA surgery. However, Osteoarthritis Research Society International (OARSI) guidelines to postpone surgery until first-line treatments for osteoarthritis are no longer helpful might be especially relevant for these patients [49].

For the secondary outcome, pain-related functional impairment five years after surgery, we found that more severe preoperative anxiety symptoms were associated with moderate to severe pain-related functional impairment, while male sex was associated with less pain-related functional impairment five years after TKA. We did not find any correlation between preoperative and postoperative pain-related functional impairment, which perhaps was surprising, as other studies, including a meta-analysis, identified a correlation between worse preoperative and postoperative function at one [18] and five years [3, 19]. This discrepancy suggests that additional factors might influence pain-related functional impairment five years after TKA, which needs to be addressed in future studies.

We found that one out of three patients had persistent pain and nearly one in five had pain-related functional impairment five years after TKA. The high incidence of persistent pain is similar to previously reported findings [50] and may reflect a complex interplay of factors [51], such as pain sensitization [51, 52]. Interestingly, in our sample, we observed a low proportion of patients with severe osteoarthritis (KL grade IV), but a high proportion of patients with more severe preoperative pain, which highlights that there may be factors other than the structural changes in the joint prior to replacement that affect pain severity after TKA. Notably, our results contrast to those from a registry study, which reported a lower incidence of persistent pain at 22%, though the incidence of functional impairment was similar at 23% [3]. Although there were no notable sample characteristics that might explain the high rate of moderate to severe pain observed in this study, there is the possibility that selection bias played a role, with patients experiencing pain at five years being more motivated to participate in this follow-up than patients without pain. It was beyond the scope of this study to explore whether pain at five years was due to a prolonged recovery period or an increase in pain over time, but a recent study suggests that there is a subgroup of patients that fluctuates in and out of a chronic pain pattern during the five-year follow-up period [8]. There is therefore a need to establish the course of recovery for non-improvers in pain and physical function, and to investigate whether there are certain preoperative characteristics that predispose these patients to adverse pain and physical function outcomes so that targeted interventions can be developed to facilitate their recovery.

### Limitations and strengths

Our study, like many longitudinal follow-up studies, encountered attrition over time. While there is no consensus on an acceptable attrition rate in prospective observational studies, Grooten et al. [53] proposed a response rate of 67% as a cut-off for attrition in their study on inter-rater agreement of risk of bias assessment in prognostic studies. In our study, the response rate exceeded this cut-off, reaching 69%, within an acceptable range according to their suggestion. Prior prospective observational studies have reported response rates of 29% [21] and 57% [20], highlighting the often low and highly variable response rates among studies with longer-term follow-up. In the current study, the respondents and non-respondents did not differ significantly on most baseline characteristics. However, patients included in the analysis had a significantly (*p* = 0.03) lower mean BMI (28.6, SD 4.2) than those lost to follow-up (mean 30.3, SD 5.6). Despite this difference, the difference was small (Cohen’s d = 0.3) and unlikely to be clinically relevant. Thus, it cannot be ruled out that the lower mean BMI of this sample may be an explanation for the fact that we did not detect an association between BMI and function at 5 years. Despite BMI being lower after 5 years, there was still sufficient variability in the variable to reveal possible association with function. We therefore believe that our results can be generalised to patients with osteoarthritis scheduled for primary TKA. Our statistical model was based on hypotheses, and we used the logistic conditional backward regression model, a recommended model in prognosis research [54]. A common pitfall is to overfit the logistic regression model. However, as we adhered to the principle with at least 100 participants, and deliberately limited the number of factors to be included, we are confident that we had enough statistical power to estimate our regression estimates with sufficient precission so that our results are valid and reliable.

In this study, the identification of evidence-based prognostic factors for pain and functional impairment post-TKA serves as a foundation for future research in developing predictive models and exploring causal relationships. These prognostic factors, once validated, could be instrumental in creating predictive models to evaluate patient outcomes and to understand the underlying causal mechanisms contributing to these outcomes after TKA [36, 54]. However, unmeasured additional factors may exist, related or unrelated to TKA, that could have influenced the outcome. Our results indicate several factors that should be considered for inclusion in future prognostic models with the aim to identify the best set of prognostic factors for predicting patients’ risk for unfavourable TKA outcomes.

While our study did not include surgical factors and post-operative complications in the modelling, it is important to acknowledge that such factors may have a significant impact on pain and functional outcomes after TKA. Future studies should explore the potential associations of these factors and include them in prognostic models to better understand their contribution to TKA outcomes.

Lastly, our primary outcome measure, the BPI, assesses pain, but might not specifically represent index joint pain. While controlling for the number of pain sites may partially address this limitation, it does not distinguish the impact of remote site pain from index joint pain. Nonetheless, the BPI is recommended for assessing persistent pain after TKA [51] as it is a reliable and validated instrument for those undergoing TKA surgery [29] and has been employed in prior studies of patients with persistent pain after TKA [55].

## Conclusion

In this study, more preoperative anxiety symptoms were associated with moderate to severe pain and pain-related functional impairment five years after TKA surgery. In addition, preoperative pain, number of painful sites, and osteoarthritis severity were factors associated with moderate to severe pain five years after TKA. Male sex was associated with less pain-related functional impairment five years after TKA. The factors identified in this study should be used to develop prognostic models for mid-term pain or pain-related functional impairment outcomes after TKA surgery.

### Electronic supplementary material

Below is the link to the electronic supplementary material.

Supplementary Material 1

## Data Availability

The datasets utilized in this study are not publicly accessible to ensure the protection of individual privacy and adhere to legal restrictions. However, upon reasonable request and with approval from the Regional Committee in Ethics in Medical Research in South-East Norway (REC) and the hospitals’ Data Protection Officer, a minimal dataset may be made available by the project leader, Maren Falch Lindberg. E-mail: marenfalch.lindberg@lds.no.

## Abbreviations

ASA: American Society of Anesthesiologists’ physical status classification
BPI: Brief Pain Inventory
BMI: Body mass index
CI: Confidence interval
HADS: Hospital Anxiety and Depression Scale (HADS)
K-L grade: Kellgren-Lawrence classification system
NRS: Numerical Rating Scale
OA: Osteoarthritis
OARSI: Osteoarthritis Research Society International
SD: Standard deviation
TKA: Total knee arthroplasty
