# Supplementary material for: Factors associated with pain and functional impairment five years after total knee arthroplasty: a prospective observational study
Source: BMC Musculoskelet Disord. 2024 Jan 2;25:22. doi: 10.1186/s12891-023-07125-y (PMC10759478; doi:10.1186/s12891-023-07125-y)
Supplement: Supplementary file 1 — Supplementary Material 1 [file 12891_2023_7125_MOESM1_ESM.doc]

STROBE Statement—Checklist of items that should be included in reports of ***cohort studies***

|  | Item No | Recommendation |
| --- | --- | --- |
| **Title and abstract** | 1 | Yes, in Abstract (*a*) Indicate the study’s design with a commonly used term in the title or the abstract |
|  |  | Yes, in abstract. (*b*) Provide in the abstract an informative and balanced summary of what was done and what was found |
| Introduction | | |
| Background/rationale | 2 | Yes, on Pg 5 Explain the scientific background and rationale for the investigation being reported |
| Objectives | 2 | Yes, on Pg 5-6 State specific objectives, including any prespecified hypotheses |
| Methods | | |
| Study design | 3 | Yes, on Pg. 7. Present key elements of study design early in the paper |
| Setting | 5 | Yes, on Pg. 7. Describe the setting, locations, and relevant dates, including periods of recruitment, exposure, follow-up, and data collection |
| Participants | 6 | Yes, on Pg. 7,8 (*a*) Give the eligibility criteria, and the sources and methods of selection of participants. Describe methods of follow-up |
|  |  | N/A. (*b*) For matched studies, give matching criteria and number of exposed and unexposed |
| Variables | 7 | Yes, on Pg. 8, 9. Clearly define all outcomes, exposures, predictors, potential confounders, and effect modifiers. Give diagnostic criteria, if applicable |
| Data sources/ measurement | 8* | Yes, on Pg. 8-10. For each variable of interest, give sources of data and details of methods of assessment (measurement). Describe comparability of assessment methods if there is more than one group |
| Bias | 9 | Yes, on Pg. 11. Describe any efforts to address potential sources of bias |
| Study size | 10 | Yes, on Pg. 10. Explain how the study size was arrived at |
| Quantitative variables | 11 | Yes, on Pg. 10,11. Explain how quantitative variables were handled in the analyses. If applicable, describe which groupings were chosen and why |
| Statistical methods | 12 | Yes, on Pg. 11. (*a*) Describe all statistical methods, including those used to control for confounding |
|  |  | N/A. (*b*) Describe any methods used to examine subgroups and interactions |
|  |  | Yes, on Pg. 10. (*c*) Explain how missing data were addressed |
|  |  | Yes, on Pg. 13. (*d*) If applicable, explain how loss to follow-up was addressed |
|  |  | Yes, on Pg. 11. (*e*) Describe any sensitivity analyses |
| Results | | |
| Participants | 13* | Yes, on Pg. 12. (a) Report numbers of individuals at each stage of study—eg numbers potentially eligible, examined for eligibility, confirmed eligible, included in the study, completing follow-up, and analysed |
|  |  | Yes, on Pg. 12. (b) Give reasons for non-participation at each stage |
|  |  | Yes, on in Additional file. A flow diagram was inserted |
| Descriptive data | 14* | Yes, on Pg. 12, 13 and Table 2 (a) Give characteristics of study participants (eg demographic, clinical, social) and information on exposures and potential confounders |
|  |  | Yes, Table 2. (b) Indicate number of participants with missing data for each variable of interest |
|  |  | Yes, on Pg. 12. Table 3. (c) Summarise follow-up time (eg, average and total amount) |
| Outcome data | 15* | Yes, Table 3. Report numbers of outcome events or summary measures over time |
| Main results | 16 | Yes, on Pg. 14-16 (Table 4 and 5). (*a*) Give unadjusted estimates and, if applicable, confounder-adjusted estimates and their precision (eg, 95% confidence interval). Make clear which confounders were adjusted for and why they were included |
|  |  | N/A (*b*) Report category boundaries when continuous variables were categorized |
|  |  | N/A (*c*) If relevant, consider translating estimates of relative risk into absolute risk for a meaningful time period |
| Other analyses | 17 | Yes, on Pg. 15. Report other analyses done—eg analyses of subgroups and interactions, and sensitivity analyses |
| Discussion | | |
| Key results | 18 | Yes, on Pg.16. Summarise key results with reference to study objectives |
| Limitations | 19 | Pg. 19-20. Discuss limitations of the study, taking into account sources of potential bias or imprecision. Discuss both direction and magnitude of any potential bias |
| Interpretation | 20 | Pg. 16-19. Give a cautious overall interpretation of results considering objectives, limitations, multiplicity of analyses, results from similar studies, and other relevant evidence |
| Generalisability | 21 | Pg. 19. Discuss the generalisability (external validity) of the study results |
| Other information | | |
| Funding | 25 | Pg. 23. Give the source of funding and the role of the funders for the present study and, if applicable, for the original study on which the present article is based |

*Give information separately for exposed and unexposed groups.
